# Evaluation of *Serratia marcescens* Adherence to Contact Lens Materials

**DOI:** 10.3390/microorganisms11010217

**Published:** 2023-01-15

**Authors:** Reed Pifer, Valerie Harris, Deaja Sanders, Monica Crary, Paul Shannon

**Affiliations:** Alcon Research, LLC, 6201 South Freeway, Fort Worth, TX 76134, USA

**Keywords:** *Serratia marcescens*, keratitis, bacterial adhesion, microbial adhesion, medical device, contact lens, poly-(2-methacryloyloxyethyl phosphorylcholine), PMPC

## Abstract

Bacterial keratitis is a risk associated with the use of contact lenses for cosmetic purposes or vision correction. In this in vitro experimental study, we examined the ability of the ocular pathogen *Serratia marcescens* to adhere to monthly or biweekly replacement contact lenses. We performed quantitative adhesion assays to evaluate the adherence of *S. marcescens* to seven contact lens materials: comfilcon A, senofilcon A, omafilcon B, fanfilcon A, balafilcon A, senofilcon C, and lehfilcon A. Lehfilcon A is a newly marketed silicon hydrogel contact lens with a surface modification of poly-(2-methacryloyloxyethyl phosphorylcholine) (PMPC). PMPC has previously been demonstrated to be an effective anti-biofouling treatment for numerous surfaces. We observed low *S. marcescens* adherence to lehfilcon A compared to other materials. We demonstrate the use of the fluorescent dye 5(6)-Carboxytetramethylrhodamine succinimidyl ester to covalently stain live cells prior to material adhesion studies.

## 1. Introduction

Microbial keratitis (MK) is an infection of the corneal epithelial layer characterized initially by pain, redness, and discharge but, in severe cases, can progress to corneal ulceration, loss of visual acuity, and even blindness. Susceptibility to the development of MK is multifactorial [1], including pathogen exposure, immune system response, corneal epithelium integrity, and patient behavior. Contact lens usage is associated with infectious keratitis, most likely through a combination of mechanisms including corneal abrasions sustained during insertion or inappropriate overnight wear and by serving as a vehicle for the introduction of pathogens into the eye [2,3]. The annual rate of MK is 2–20 cases per 10,000 contact lens wearers [1]. It is believed that poor contact lens hygiene practices by patients is a major contributing factor for MK [4,5,6,7,8,9,10].

The normal ocular flora of healthy individuals is composed of a low density of multiple bacterial species, particularly the Gram-positive species *Corynebacterium* spp., *Staphylococci* spp., *Streptococcus* spp., *Propionibacterium* spp., and *Micrococcus* spp. [11,12,13,14,15,16], and the Gram-negative species *Acinetobacter* spp. and *Sphingomonas* spp. [15], whose alteration is associated with disease [17]. Contact lens wear is associated with alterations to the eye microbiota [18]. *Serratia* spp. are not thought to be stable members of the healthy ocular microbiota; however, *S. marcescens* is commonly isolated from corneal scrapings and contact lenses of patients experiencing contact lens-related microbial keratitis [2,3,19,20]. *S. marcescens* is associated with corneal ulceration [3,21,22] and expresses multiple protease virulence factors that enable corneal tissue destruction [23,24]. Treatment of ocular *Serratia* spp. infections routinely involves topical antibiotics, typically fluoroquinolones such as ciprofloxacin or aminoglycosides such as tobramycin [22]. These ocular infections may be enabled by the reduced disinfection efficacy of some contact lens care solutions for clinical strains of *S. marcescens* [23,25]. Indeed, *S. marcescens* has been found in the contact lens storage cases of both symptomatic and asymptomatic individuals [3,8].

In a prospective study, Dart et al. performed a microbial keratitis risk analysis of patient behaviors and contact lens brands then available [7]. The authors observed differences in the keratitis risks across brands of contact lenses, suggesting that contact lens polymer choice may play an important role in mitigating microbial keratitis. Substantial research effort has been dedicated to improving medical device polymers to reduce microbial contamination. The contact lens field has focused on modifying the base material of polymers with a variety of anti-biofouling formulations, including antimicrobial peptides [26,27,28,29,30], silver [31,32], and quorum sensing inhibitors [33,34].

In this in vitro experimental study, we evaluated the ability of a clinical isolate of *S. marcescens* to adhere to a variety of monthly or biweekly replacement contact lens materials. The materials tested are comfilcon A, senofilcon A, omafilcon B, fanfilcon A, balafilcon A, senofilcon C, and lehfilcon A. Lehfilcon A is a newly marketed silicon hydrogel contact lens with a surface modification containing poly-(2-methacryloyloxyethyl phosphorylcholine) (PMPC) [35,36]. MPC has been described to impart anti-biofouling properties to numerous base substrates [36,37,38,39,40,41,42,43].

## 2. Materials and Methods

**Strains, Growth Conditions, and Materials**. The *S. marcescens* strains MCC 7246 and MCC 7239 were isolated from corneal scrapings of keratitis patients and obtained from Ciba Vision. The pond water isolate ATCC 13880, routinely used for quality control testing, was obtained from the American Type Culture Collection. *S. marcescens* strains were routinely cultured and maintained on trypticase soy agar (TSA) medium incubated overnight at 30–35 °C for 16–24 h. Starting inocula for broth cultures were prepared from fresh overnight TSA slants resuspended in 0.9% NaCl. Cell density was adjusted to approximately 1 × 10^8^ CFU/mL by volumetric addition until an appropriate percent transmittance at 525 nm was reached using a Thermo Spectronic 20D+ spectrophotometer. Broth cultures were prepared by dilution of the starting inoculum to approximately 1 × 10^7^ CFU/mL in culture medium. Bacteria were enumerated by serial dilution into PBST, and dilutions were plated in duplicate using TSA supplemented with 0.5% polysorbate 80 and 0.07% lecithin (MCTA). Colonies were counted following incubation at 30–35 °C for 16–24 h. Experiments were performed using trypticase soy broth (TSB) or Neidhardt’s MOPS Minimal Media (Teknova) [44,45], supplemented to 10% with heat-inactivated fetal bovine serum (FBS). All broth cultures were incubated at 30–35 °C at 100 RPM in a New Brunswick Innova 40R shaking incubator, unless otherwise stated.

Experiments were performed on commercially purchased contact lenses. The following contact lens types were used: Alcon TOTAL30™ (lehfilcon A), CooperVision^®^ Biofinity^®^ (comfilcon A), CooperVision^®^ Proclear^®^ (omafilcon B), CooperVision^®^ Avaira Vitality^®^ (fanfilcon A), Johnson & Johnson Vision Care Acuvue^®^ Oasys (senofilcon A), Johnson & Johnson Vision Care Acuvue^®^ Vita™ (senofilcon C), and Bausch + Lomb Purevision^®^ (balafilcon A). To minimize the potential confounding antimicrobial activities of packaging saline on experimental results, all lenses were equilibrated in purified water prior to experiments. Contact lenses were aseptically removed from their primary packaging using sterile plastic forceps and transferred into 12-well polystyrene plates containing 3 mL of purified water per well. Contact lenses were shaken at 100 RPM for 5 min and transferred to wells containing fresh, purified water. A total of three 5 min rinses were performed per contact lens. Contact lenses were then soaked overnight at 100 RPM and 30–35 °C for 16–24 h in purified water. 

**Adhesion Assays**. Quantitative adhesion assays were performed to assess contact lens material binding by *S. marcescens* MCC 7246, MCC 7239, and ATCC 13880. At a volume of 1.5 mL/well, 12-well plates were populated with an inoculated broth culture. One equilibrated contact lens per well was submerged with the concave side up and ensured to be free floating within the cell suspension. *S. marcescens* was allowed to adhere to the contact lenses for 4 h with incubation at 30–35 °C and 100 RPM. To remove non-adherent bacteria, lenses were gently transferred by sterile forceps into 12-well plates containing 3 mL of Phosphate Buffered Saline (PBS) and agitated at 100 RPM for 5 min at 30–35 °C. Five sequential rinses were performed per contact lens. Contact lenses were transferred into tubes containing 10 mL of PBS supplemented with 0.05% polysorbate 80 (PBST). Bacteria were eluted from the lenses through vortexing and sonication using a VWR Aquasonic sonicator. Bacteria were enumerated as described above.

**Statistical Analysis**. The surface area in square millimeters of each contact lens was determined using the following formula:

A = 2π [(D/2)^2^ + S^2^] (1)

where D indicates the lens diameter. S refers to lens posterior sag. S is calculated using the following formula, where BCE refers to the base curve:

S = BCE − √(BCE^2^ − (D/2)^2^). (2)

Duplicate growth plate count enumerations were averaged, and the CFU/mL was calculated for the organism suspension containing the contact lens. To calculate the CFU/lens, the CFU/mL value was multiplied by 10 to account for the elution volume. The microbial density of a contact lens was determined by dividing the number of organisms per lens by the surface area of the contact lens (CFU/mm^2^). The log_10_ microbial density (log CFU/mm^2^) was calculated for each lens. 

GraphPad Prism version 9 was used for statistical analysis. To determine if the microbial recovery was significant, a one-way ANOVA was used to compare the log microbial densities recovered from each contact lens material. Bonferroni’s post-hoc multiple comparison tests were performed to compare lehfilcon A to other materials. A *p*-value of 0.05 was considered significant. The number of replicates used for each experiment is reported in each figure legend, and the *p*-values of specific comparisons are reported in the results section.

**Fluorescent staining**. Freshly prepared overnight TSA slants of MCC 7246 were used to inoculate 3 mL TSB broth cultures. After 16–24 h of growth as previously described, broth cultures were centrifuged at 5000 RPM in an Eppendorf 5424 Centrifuge for 5 min. Cell pellets were rinsed twice in 1 mL of PBS. In DMSO, stock solutions of 5-(and-6)-Carboxytetramethylrhodamine N-succinimidyl ester (TAMRA-SE; Invitrogen) of 10 mg/mL were prepared. Cell pellets were resuspended in 1 mL PBS containing 100 mg/mL TAMRA-SE and incubated for 10 min at 30–35 °C. Stained cells were pelleted and rinsed in PBS for 5 sequential 1 mL rinses to remove unreacted TAMRA stain prior to performing adhesion assays. Broth cultures of TAMRA-stained *S. marcescens* were prepared by dilution to approximately 1 × 10^7^ CFU/mL and used in the adhesion assay as described above.

**Confocal Microscopy**. Fluorescent imaging of TAMRA-stained *S. marcescens* MCC 7246 adherence to the surface of contact lenses was performed in a modified adhesion assay. Following 4 h of contact time between contact lenses and *S. marcescens*, lenses were gently transferred into wells containing 3 mL of 4% paraformaldehyde in PBS and fixed for 5 min at 30–35 °C and 100 RPM. Following fixation, lenses were rinsed four times in wells containing 3 mL PBS. Rinsed lenses were mounted for imaging in Prolong Live antifade agent diluted into PBS according to the manufacturer’s instructions. Samples were imaged using a Nikon A1R confocal microscope with a 561 nm excitation laser, a 570–616 nm band pass filter, and a 10X objective at a 4.975 micrometer resolution. Z-stacks were collected from the central portion of colonized or uncolonized control lenses, and single-layer images were obtained as maximum intensity projections. Histograms of raw pixel intensity were generated in GraphPad Prism from frequency data collected in Nikon Elements version 5.30.05. For whole-lens microscopy, large image stitching and composite 3D renderings of images were performed in Nikon Elements.

## 3. Results

**Figure 1:**
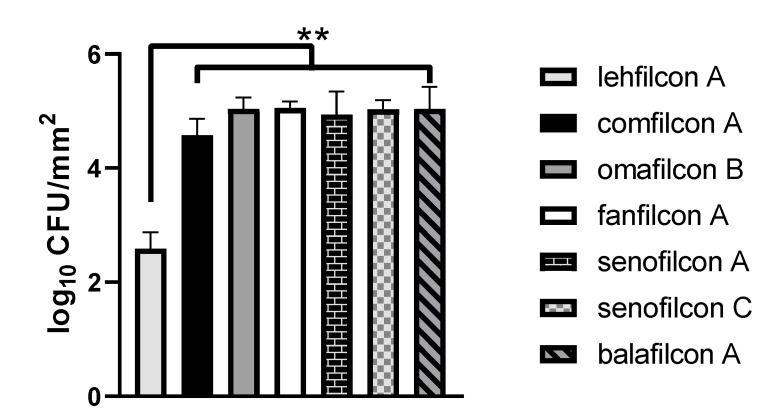
*S. marcescens* demonstrates low adhesion to lehfilcon A relative to other contact lens materials. Adhesion assays of *S. marcescens* MCC 7246 exposed to soft contact lenses (*n* = 8 per lens material) in TSB medium containing 10% FBS as a soiling agent. The graph depicts the average log density of cells recovered after adhesion reactions (log CFU/mm^2^ ± S.D.). ** indicates *p* < 0.0001 for comparison to lehfilcon A.

To evaluate the propensity of each material to allow *S. marcescens* adhesion, we performed quantitative adhesion assays in which *S. marcescens* was briefly cultured in contact with each contact lens material. After culturing and gentle rinsing of each lens, the bacteria were eluted from each lens, and viable organisms were enumerated. We witnessed in Figure 1 a 2.0 log adhesion advantage of lehfilcon A (2.6 ± 0.3 log CFU/mm^2^, mean ± S.D.) relative to comfilcon A (4.6 ± 0.3 log CFU/mm^2^; *p* < 0.0001), a 2.4 log advantage relative to omafilcon B (5.0 ± 0.2 log CFU/mm^2^; *p* < 0.0001), a 2.5 log advantage relative to fanfilcon A (5.1 ± 0.1 log CFU/mm^2^; *p* < 0.0001), a 2.3 log adhesion advantage relative to senofilcon A (4.9 ± 0.4 log CFU/mm^2^; *p* < 0.0001), a 2.4 log advantage relative to senofilcon C (5.0 ± 0.2 log CFU/mm^2^; *p* < 0.0001), and a 2.4 log advantage relative to balafilcon A (5.0 ± 0.4 log CFU/mm^2^; *p* < 0.0001). 

The low bacterial density observed on lehfilcon A (Figure 1) is expected given that MPC modifications also reduce bacterial adhesion to silicone interocular lenses [37]. Notably, lehfilcon A outperformed omafilcon B, which also includes MPC in its formulation. The PMPC constituent of lehfilcon A is found within a surface-exposed layer on top of a silicone hydrogel base material [35,36], whereas omafilcon B is composed of MPC crosslinked with 2-hydroxyethylmethacrylate (HEMA). We are unaware of studies relating to the solvent accessibility of MPC on omafilcon B. Previous studies regarding protein adsorption by the MPC-containing omafilcon A contact lens material did not suggest that the material outperformed other HEMA-based materials, such as etafilcon A [46].

We aimed to confirm the quantitative adhesion results found in Figure 1 with a qualitative microscopy study of the same materials. We designed a covalent cell surface fluorescent stain using the rhodamine derivative TAMRA-SE to facilitate fluorescent microscopy. However, we observed substantial autofluorescence of the contact lenses, attributable in part to the rich TSB media used for the adhesion assay method. Therefore, we evaluated the adhesion profile of *S. marcescens* in a low fluorescence minimal media based upon Neidhardt’s MOPS minimal medium (MOPS). MOPS has been used to cultivate *S. marcescens* [44], as well as a variety of other Gram-negative pathogens [45,47,48,49]. 

**Figure 2:**
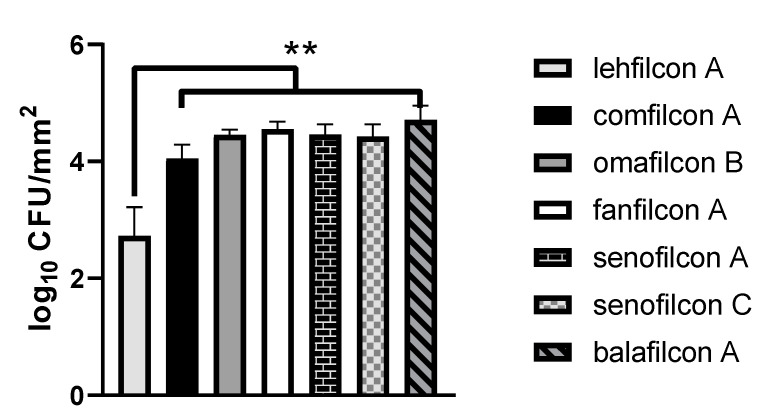
*S. marcescens* demonstrates low relative adhesion to lehfilcon A in low autofluorescence media. Adhesion assays of *S. marcescens* MCC 7246 exposed to soft contact lenses (*n* = 8 per lens material) in MOPS minimal media supplemented with 10% FBS. The graph depicts the average log density of cells recovered after adhesion reactions (log CFU/mm^2^ ± S.D.). ** indicates *p* < 0.0001 for comparison to lehfilcon A.

We found that the trend of *S. marcescens* adhesion to the contact lens materials tested is maintained in the minimal media formulation (Figure 2). Specifically, we witnessed a 1.3 log average adhesion advantage of lehfilcon A (2.7 ± 0.5 log CFU/mm^2^) relative to comfilcon A (4.1 ± 0.3 log CFU/mm^2^; *p* < 0.0001), a 1.8 log average advantage relative to omafilcon B (4.5 ± 0.1 log CFU/mm^2^; *p* < 0.0001), a 1.8 log average advantage relative to fanfilcon A (4.5 ± 0.1 log CFU/mm^2^; *p* < 0.0001), a 1.8 log average adhesion advantage relative to senofilcon A (4.5 ± 0.2 log CFU/mm^2^; *p* < 0.0001), a 1.7 log average advantage relative to senofilcon C (4.4 ± 0.2 log CFU/mm^2^; *p* < 0.0001), and a 2.0 log average advantage relative to balafilcon A (4.7 ± 0.2 log CFU/mm^2^; *p* < 0.0001). 

**Figure 3:**
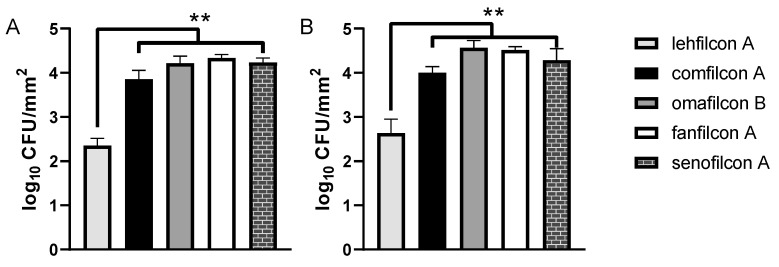
Fluorescent surface staining of live *S. marcescens* does not affect contact lens adhesion trends. (**A**) Adhesion assay using *S. marcescens* MCC 7246 left unstained prior to exposure to soft contact lenses (*n* = 6 per lens type) in MOPS minimal media containing 10% FBS. (**B**) Adhesion assay using *S. marcescens* MCC 7246 stained with TAMRA-SE prior to exposure to soft contact lenses (*n* = 6 per lens type) in MOPS minimal media containing 10% FBS. Graphs depict the average log density of cells recovered after adhesion (log CFU/mm^2^ ± S.D.). ** indicates *p* < 0.0001 for comparison to lehfilcon A.

The TAMRA-SE staining procedure presumably relies on the formation of covalent bonds between the primary amine residues of surface-expressed proteins and the succinimidyl group of the dye. Bacterial adhesion to contact lenses is dependent upon a multitude of variables; however, modification of cell surface proteins has the potential to alter the binding characteristics of bacteria. Therefore, we evaluated the effect of TAMRA-SE staining on *S. marcescens* adhesion to a subset of contact lens materials. We observed no difference in the adhesion profiles of contact lens materials bound by unstained (Figure 3a) or cell surface-stained *S. marcescens* (Figure 3B). 

**Figure 4:**
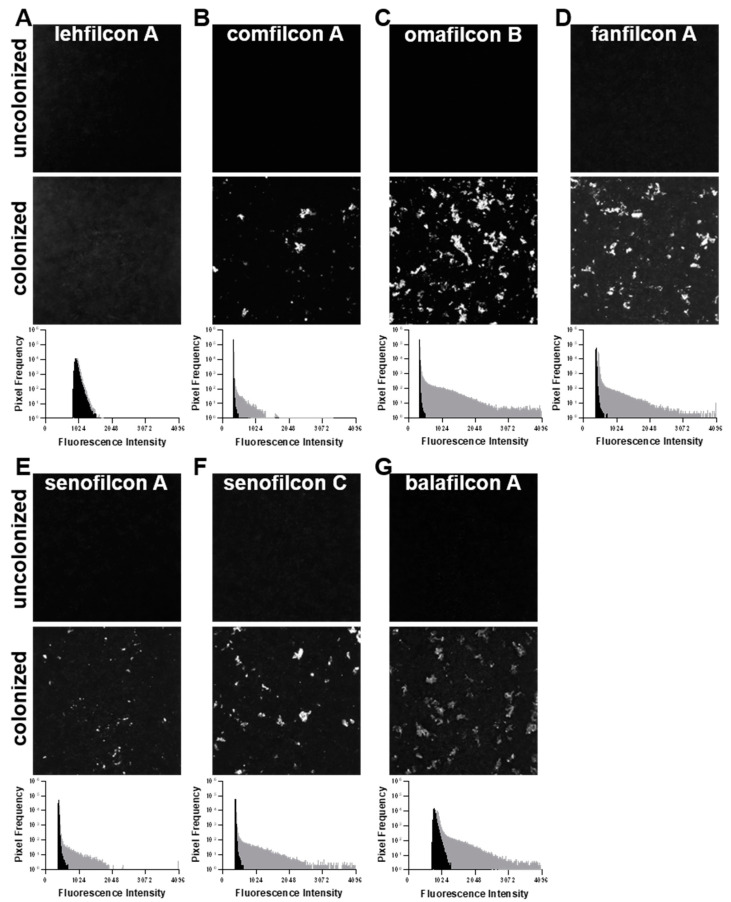
Confocal microscopy of contact lenses colonized with TAMRA-stained *S. marcescens*. Image size of 0.42 mm × 0.42 mm taken from the central region of lehfilcon A (**A**), comfilcon A (**B**), omafilcon B (**C**), fanfilcon A (**D**), senofilcon A (**E**), senofilcon C (**F**), and balafilcon A (**G**) was collected at 10X magnification. Uncolonized control contact lenses (top panel) and *S. marcescens*-colonized contact lenses (middle panel) are shown for comparison. Histograms (bottom panel) depict the frequency distribution of fluorescent pixel intensities from uncolonized (black) and colonized (gray) contact lenses.

**Figure 5:**
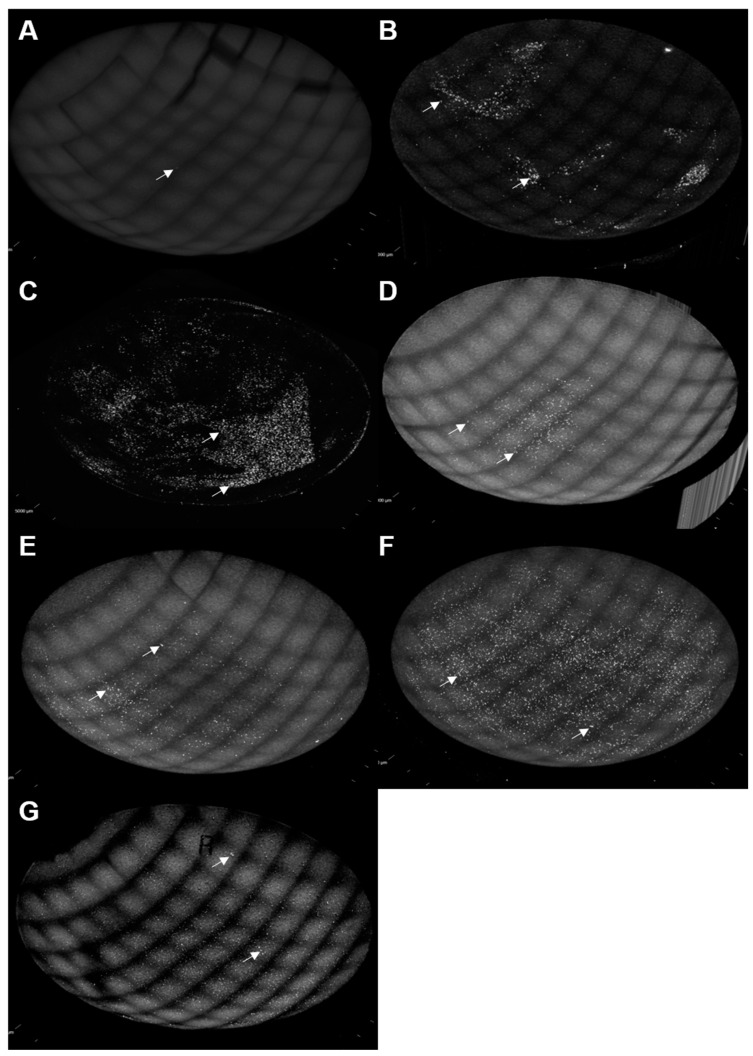
Whole lens confocal microscopy of contact lenses colonized with TAMRA-stained *S. marcescens*. Composite whole-lens images of lehfilcon A (**A**), comfilcon A (**B**), omafilcon B (**C**), fanfilcon A (**D**), senofilcon A (**E**), senofilcon C (**F**), and balafilcon A (**G**) were stitched from fields collected at 10× magnification. Arrows indicate examples of foci of *S. marcescens* adhered to the contact lens surfaces.

Using TAMRA-labeled *S. marcescens*, we qualitatively assessed adherence to the contact lens materials by confocal microscopy (Figure 4 and Figure 5). Figure 4 depicts images taken from the center of contact lenses colonized with fluorescent *S. marcescens* or uncolonized controls. We observed apparent clusters of bacteria visible on comfilcon A, omafilcon B, fanfilcon A, senofilcon A, senofilcon C, and balafilcon A upon colonization. According to our quantitative analyses, lehfilcon A has few colonizing bacteria, as shown in Figure 1 and Figure 2.

Figure 5 depicts whole lens composite images for each lens material quantitatively evaluated in Figure 1 and Figure 2. Bacteria are seen as brighter-than-background foci on each lens. We observed low relative binding to lehfilcon A (Figure 5A), an intermediate amount of binding to comfilcon A (Figure 5B), and high binding to omafilcon B (Figure 5C), fanfilcon A (Figure 5D), senofilcon A (Figure 5E), senofilcon C (Figure 5F), and balafilcon A (Figure 5G). The background autofluorescence differs between lenses, with comfilcon A (Figure 5B) and omafilcon B (Figure 5C) having very low autofluorescence. A waffle pattern can be seen in most images that results from the stitching procedure used to generate the whole lens composite images. In Figure 1 and Figure 2, a trend towards lower-than-average bacterial adhesion can be seen for comfilcon A. This trend is recapitulated in Figure 4B, as comfilcon A tends to accumulate patches of dense bacteria surrounded by low-density areas rather than allowing for homogenous colonization as seen with other materials.

**Figure 6:**
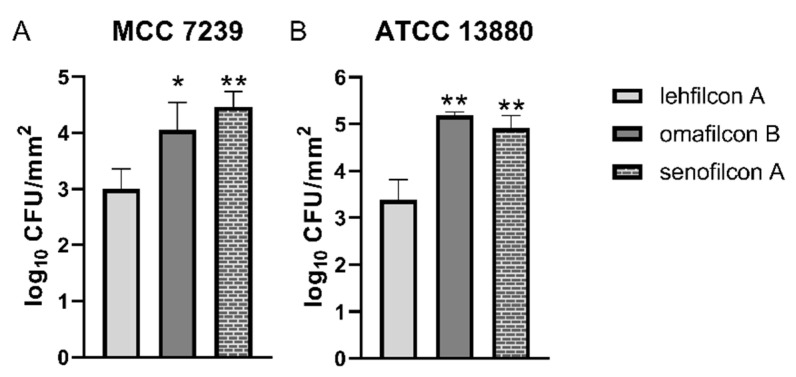
The phenomenon of low adhesion to lehfilcon A generalizes across *S. marcescens* strains. Adhesion assay using (**A**) *S. marcescens* strain MCC 7239 or (**B**) strain ATCC 13880 exposed to soft contact lenses (*n* = 6 per lens material) in TSB medium containing 10% FBS as a soiling agent. Graphs depict the average log density of cells recovered after adhesion reactions (log CFU/mm^2^ +/− S.D.). * indicates *p* < 0.001 and ** indicates *p* < 0.0001 for comparisons to lehfilcon A.

To determine whether the trend of low relative adhesion to lehfilcon A extends to other strains of *S. marcescens*, we performed quantitative adhesion assays for two additional strains, MCC 7239 and ATCC 13880 (Figure 6). We observed that MCC 7239 adhered to lehfilcon A (3.0 ± 0.4 log CFU/mm^2^) at a lower level than to the MPC-containing material omafilcon B (4.0 ± 0.5 log CFU/mm^2^, *p* = 0.0001) or the silicone hydrogel material of senofilcon A (4.5 ± 0.3 log CFU/mm^2^, *p* < 0.0001). Similarly, we witness low binding by ATCC 13880 to lehfilcon A (3.4 ± 0.4 log CFU/mm^2^) relative to omafilcon B (5.2 ± 0.1 log CFU/mm^2^, *p* < 0.0001) and senofilcon A (4.9 ± 0.3 log CFU/mm^2^, *p* < 0.0001). These results suggest that the PMPC-modified surface of lehfilcon A produces an anti-biofouling effect that does not depend upon the *S. marcescens* strain used for testing.

## 4. Discussion

Contact lens users regularly develop non-compliant behaviors that compromise the ability of contact lens care solutions to effectively control microbiological contamination risks. “Topping off” is the practice of adding only a small volume of contact lens care solution to a contact lens case containing a preexisting volume of spent solution. This action satisfies the consumer’s desire to fill the contact lens case to the recommended fill line (superficial compliance) while unintentionally diluting the available biocides within the contact lens care solution (effective non-compliance) [50,51]. Multiple authors have described contact lens storage cases as a reservoir of potential pathogens [3,8,52,53]. Similarly, patients routinely fail to perform “rub and rinse” regimens for their contact lenses, despite recommendations by contact lens care solution manufacturers [51]. These patterns of behavior persist despite patients being informed of proper practices by their eye care professionals [50]. As a result, contact lenses may serve as a vehicle to introduce potential pathogens into a patient’s eye. New polymer technologies for contact lenses and contact lens storage cases could be a promising future avenue for mitigating the effects of patient noncompliance by providing an additional layer of biocontamination control on top of that provided by contact lens care solutions [54,55]. 

The development of new medical technologies involves the collaborative efforts and mutual understanding of many disciplines. Microscopy-based imaging analyses are important for gaining a mechanistic understanding of microbial interactions with medical devices. However, in our experience, the greatest impact of microscopy methods is their unique capacity to generate communicable insights that traverse the barriers between fields of expertise. Unlike lab-adapted model strains of pathogenic organisms, clinical isolates usually lack any genetic tools that would enable studies that use fluorescent proteins such as green fluorescent protein. Therefore, externally applied stains are needed to facilitate microscopic analysis of bacterial interactions with medical devices. While traditional fluorescent stains such as Nile Red, Acridine Orange, or SYTO 9 are readily available, these dyes have downsides including photobleaching, macromolecule binding-dependent fluorescence, and susceptibility to loss via microbial drug efflux pumps. In this study, we used an impermeable fluorescent succinimidyl ester to covalently label the cell surface of live cells, as described by Fuller et al. [56]. An obvious advantage of succinimidyl ester stains is that they can be readily acquired in combination with many fluorophores. We demonstrated the use of 5(6)-Carboxytetramethylrhodamine succinimidyl ester to stain the *S. marcescens* clinical isolate MCC 7246 prior to microscopy evaluation of contact lens adhesion (Figure 4 and Figure 5). 

We observed that TAMRA surface-stained *S. marcescens* demonstrated qualitatively low adhesion to lehfilcon A relative to other contact lens materials (Figure 4 and Figure 5). However, this pattern of *S. marcescens* adhesion was not dependent upon TAMRA staining (Figure 3), suggesting that this staining methodology could be generally useful for medical device investigations. Quantitative assays revealed a substantially lower level of *S. marcescens* adhesion to the PMPC-modified surface of lehfilcon A compared to traditional soft contact lenses (Figure 1), and this pattern was maintained across media conditions (Figure 2) and across strains (Figure 6). These results agree with prior reports of the anti-biofouling properties of other MPC-modified medical devices and test surfaces, including stainless steel [38,39], dental resins [40,41,42], absorbable sutures [43], and for a variety of organisms, including *S. aureus* [38,43], *S. epidermidis* [38], *P. aeruginosa* [38], *S. mutans* [39,41], *P. gingivalis* [39], and *Candida spp.* [40,42].

## Data Availability

Not applicable.
